# Narcolepsy—A Neuropathological Obscure Sleep Disorder: A Narrative Review of Current Literature

**DOI:** 10.3390/brainsci12111473

**Published:** 2022-10-30

**Authors:** Vishal Chavda, Bipin Chaurasia, Giuseppe E. Umana, Santino Ottavio Tomasi, Bingwei Lu, Nicola Montemurro

**Affiliations:** 1 Department of Pathology, Stanford of School of Medicine, Stanford University Medical Centre, Palo Alto, CA 94305, USA; 2 Department of Neurosurgery, Neurosurgery Clinic, Birgunj 44300, Nepal; 3 Department of Neurosurgery, Associate Fellow of American College of Surgeons, Trauma and Gamma-Knife Centre, Cannizzaro Hospital Catania, 95100 Catania, Italy; 4 Department of Neurological Surgery-Christian Doppler Klinik, 5020 Salzburg, Austria; 5 Department of Neurosurgery, Azienda Ospedaliera Universitaria Pisana (AOUP), University of Pisa, 56100 Pisa, Italy

**Keywords:** cataplexy, hypocretin, narcolepsy, sleep cycle disorder, sleep disorder

## Abstract

Narcolepsy is a chronic, long-term neurological disorder characterized by a decreased ability to regulate sleep–wake cycles. Some clinical symptoms enter into differential diagnosis with other neurological diseases. Excessive daytime sleepiness and brief involuntary sleep episodes are the main clinical symptoms. The majority of people with narcolepsy experience cataplexy, which is a loss of muscle tone. Many people experience neurological complications such as sleep cycle disruption, hallucinations or sleep paralysis. Because of the associated neurological conditions, the exact pathophysiology of narcolepsy is unknown. The differential diagnosis is essential because relatively clinical symptoms of narcolepsy are easy to diagnose when all symptoms are present, but it becomes much more complicated when sleep attacks are isolated and cataplexy is episodic or absent. Treatment is tailored to the patient’s symptoms and clinical diagnosis. To facilitate the diagnosis and treatment of sleep disorders and to better understand the neuropathological mechanisms of this sleep disorder, this review summarizes current knowledge on narcolepsy, in particular, genetic and non-genetic associations of narcolepsy, the pathophysiology up to the inflammatory response, the neuromorphological hallmarks of narcolepsy, and possible links with other diseases, such as diabetes, ischemic stroke and Alzheimer’s disease. This review also reports all of the most recent updated research and therapeutic advances in narcolepsy. There have been significant advances in highlighting the pathogenesis of narcolepsy, with substantial evidence for an autoimmune response against hypocretin neurons; however, there are some gaps that need to be filled. To treat narcolepsy, more research should be focused on identifying molecular targets and novel autoantigens. In addition to therapeutic advances, standardized criteria for narcolepsy and diagnostic measures are widely accepted, but they may be reviewed and updated in the future with comprehension. Tailored treatment to the patient’s symptoms and clinical diagnosis and future treatment modalities with hypocretin agonists, GABA agonists, histamine receptor antagonists and immunomodulatory drugs should be aimed at addressing the underlying cause of narcolepsy.

## 1. Introduction

Narcolepsy is a chronic, life-long neurological disorder that interferes with a person’s daily sleep cycle and wakefulness. It is primarily influenced by the REM and NREM sleep cycles and has been linked to a variety of neurological disorders [1]. It is distinguished by episodic daytime sleepiness and nighttime wakefulness, as well as sleeping difficulty. It is characterized by abnormally rapid eye movement, cataplexy and mild muscular weakness, all of which lead to body collapse or paralysis [2,3,4]. Narcolepsy affects people of all ages, including children, adolescents, adults and the elderly, and symptoms range from mild to severe. A person with narcolepsy may fall asleep at any time, such as while conversing or driving. Narcolepsy is also referred to as hypersomnia, which is a chronic REM sleep disorder characterized by excessive daytime sleepiness [3,4,5]. An individual enters the premature stage of sleep in a classic sleep cycle, followed by depth sleep stages for 90 min, where the end of REM sleep occurs. In contrast, narcolepsy patients can enter REM sleep within 15 min of starting their sleep cycle during the day [1]. REM sleep causes daydreaming and muscular paralysis in this way. Narcolepsy and its associated pathology are caused by a variety of factors [1]. Traumatic brain injury, such as stroke, injury to the hypothalamus, and loss of hypocretin receptors, underlying neurological complications such as major depressive disorders and schizophrenia, a metabolic disorder such as diabetes, and other factors can all contribute to the development of narcolepsy. The exact cause and pathophysiology of narcolepsy pathology are unknown, but it has been classified into three major types based on research and clinical experience: (1) narcolepsy accompanied by cataplexy; (2) narcolepsy without cataplexy, characterized by daytime sleepiness; and (3) secondary narcolepsy, caused by hypothalamic injury and an imbalance in neuronal transmission [6,7]. The American Academy of Sleep Medicine Board of Directors published the International Classification of Sleep Disorders, 3rd edition (ICSD-3) in 2014. In the third edition, the most drastic change in the content was the unification of secondary insomnia categories into a single “chronic insomnia” category. In the central disorders of hypersomnolence section, the nomenclature for narcolepsy was changed to narcolepsy type 1 and type 2 [8]. The nosology of narcolepsy has also been revised, subdividing the disorder into type 1 and type 2 narcolepsy, replacing narcolepsy with and without cataplexy, respectively. This reflects a change in focus from diagnosis based on symptoms to diagnosis based on pathophysiology, in this case hypocretin (orexin) deficiency status. This change was predicated on the notion that almost all patients with cataplexy have hypocretin deficiency. In addition, “narcolepsy with cataplexy” is improper because some patients with hypocretin deficiency do not have cataplexy or have yet to develop cataplexy [8]. As an increased number of studies into narcolepsy treatment options were performed in the last decade, to facilitate the diagnosis and treatment of sleep disorders and to better understand the neuropathological mechanisms of these sleep disorders, we summarized in this review all current knowledge on narcolepsy, in particular, genetic and non-genetic associations of narcolepsy, the pathophysiology up to the inflammatory response, the neuromorphological hallmarks of narcolepsy, and possible links with other diseases, such as diabetes, ischemic stroke, and Alzheimer’s disease. This review also reports all of the most recent updated research and therapeutic advances in narcolepsy.

## 2. Epidemiology

Several research studies have been conducted to determine the exact prevalence of narcolepsy, which was discovered to be between 25 and 50 per 100,000 people in Europe, Japan, and the United States [9,10,11]. As a result, narcolepsy is a rare condition. Because of a lack of specific diagnosis and symptoms that mimic other diseases, the exact epidemiology is unknown. Narcolepsy is estimated to affect between 50 and 67 per 100,000 people in North America, Western Europe and Asia [12]. The prevalence of narcolepsy is very low in India and other Asian countries, and very few cases have been reported to confirm narcolepsy with a thorough investigation. In India, the prevalence is very low because cases are not properly diagnosed, are misdiagnosed or are only partially diagnosed to confirm narcolepsy [13]. Because of the mixed symptoms and other associated neurological diseases, it is frequently misdiagnosed as seizures or obstructive sleep apnea, and thus the exact prevalence of narcolepsy worldwide is thought to be low. However, if every patient is examined and diagnosed with a differential diagnosis, the true prevalence will be greater than the reported one [14].

## 3. Genetic and Non-Genetic Associations with Narcolepsy

Despite the fact that 2–3% of familial cases are reported, narcolepsy is primarily a sporadic disorder. In different families with familial narcolepsy, an autosomal dominant mode of inheritance and incomplete penetrance with a heterogeneously single gene mutation have been observed [15]. Patients’ first-degree relatives have a 10–40 times higher risk of developing narcolepsy than the general population [16]. Identifying defective genes, on the other hand, can provide critical information about pathological mechanisms (e.g., the type 2 hypocretin receptor mutation reported in autosomal recessive narcolepsy in dogs).

### 3.1. HLA Genes: HLADQB1 as Risk Factor of Narcolepsy Type 1

Sporadic narcolepsy, like most complex diseases, is thought to be the result of a complex interaction of environmental factors and several gene variants. HLA association was first reported as a result of genetic studies to help explain the pathogenesis of narcolepsy. The HLA locus on chromosome 6 encodes the HLA complex, which is divided into three sub-regions based on their function: HLA class I, II, and III. The HLA complex is critical in the recognition and processing of foreign antigens by the immune system. HLA classes I and II encode glycoproteins involved in antigen processing and presentation to cytotoxic and helper T lymphocytes, respectively. HLA class I molecules can be found on the surface of nearly all human cells, whereas HLA class II molecules can be found on the surface of antigen-presenting cells such as B cells, macrophages, dendritic cells, and so on. HLA class I molecules, such as HLA-A, HLA-B, and HLA-C, present antigenic peptides to T-cell receptors (TCR) on CD8+ T cells, whereas HLA class II molecules, such as HLA-DR, HLA-DQ, and HLA-DP, present antigenic peptides to CD4+ T cells. HLA-DRB1, DQA1, and DQB1 are vulnerable genes associated with autoimmune diseases such as Graves’ disease [17], rheumatoid arthritis [18], type I diabetes [19,20], and narcolepsy [19,21]. As immunological tolerance to the self has evolved to protect against autoimmunity, the ability to bind and present processed antigens via HLA molecules is critical for developing self-tolerance in the thymus, primarily by removing self-reactive lymphocytes and preventing inflammatory tissue-destructive reactions. As a result, HLA alleles are linked to a wide range of autoimmune diseases.

In Japan, Europe and the United States, most narcoleptic patients have a positive correlation with the HLA-DR2 haplotype (encoding HLA class II molecules). Surprisingly, precise alleles at four linked genes (HLA-DRB5*01:01, DRB1*15:01, DQA1*01:02 and DQB1*06:02) explain susceptibility haplotype in white narcoleptic patients. This link is thought to be required for the emergence of narcolepsy, but its utility as a screening or diagnostic marker is limited by the fact that approximately 12–38 percent of the healthy, general population also carries the associated HLA haplotype, so it cannot be considered an absolute risk factor for narcolepsy [22]. Consequently, the DQB1*06:02 allele is the most common predisposing allele, appearing in at least 98 percent of cataplexy narcolepsy patients. As a consequence, HLA-DQB1*06:02 in both homozygous and heterozygous patients accounts for the variation in narcolepsy risk. The interdependent heterogeneous allele of the DQB1 locus represents a relative risk in heterozygotic individuals, whereas homozygosity is associated with a two-fold increase in risk. Other DR and DQ alleles have protective or predisposing effects in other HLA-related disorders, and the HLA association is complex with clear implications. As a result, when considering a plethora of HLA alleles, large populations are required to measure individual allelic contributions. New protective alleles DQB1*06:03, DQB1*05:01, DQB1*06:09 and DQB1*02 have recently been identified. More than 85% of narcolepsy with cataplexy patients have HLA DQB1*0602, which is frequently linked to HLA DRB1*1501, whereas only about 40% of atypical, mild narcolepsy patients without cataplexy have HLA DQB1*0602, indicating greater heterogeneity in narcolepsy without cataplexy [23]. Other HLA alleles can also influence the proclivity to narcolepsy with cataplexy. Cataplexy patients who do not have HLA DQB1 have a very rare form of narcolepsy. These findings strongly suggest that the DQB1 locus is responsible for a significant portion of the narcolepsy-related genetic risk and protection. HLA typing’s sensitivity, specificity, and discriminatory power are all low, making it unsuitable as a routine diagnostic test [16]. HLA class II antigens, particularly DQ1 and DR2, are strongly linked to narcolepsy. DQB1*06:02 and DQB1:02:00 alleles code for amino acids serine at 182 positions (DQB1Ser182) and threonine at 185 positions (DQB1Thr185), respectively, and DQB1*03:05 and DQB1*03:01 alleles code for amino acids asparagine at 182 positions (DQB1Aspar182) (DQB1Asn182). This discovery demonstrates that DQB1Ser182 and DQB1Thr185 alleles are vulnerable to the development of narcolepsy, whereas DQB1Asn182 is protective [24].

### 3.2. Non-HLA Genetic Associations

The development of narcolepsy with cataplexy is linked to molecules that are associated with MHC proteins or that regulate autoimmunity, according to genome-wide association studies (GWAS) [25]. Aside from HLA loci, narcolepsy with cataplexy is linked to the TRA and TRB loci, which both encode T cell receptor chains [26]. The allele in the joining segment, specifically within the J24 segment, that changes the amino acid phenylalanine to leucine at the complementarity determining region 3 (CDR3) peptide binding region of the T cell receptor is in linkage disequilibrium, a phenomenon that refers to the non-random association of alleles at various loci with an allele in the joining segment, specifically within the J24 segment, that alters the amino acid phenylalanine [27]. Other antigen-presenting pathway genes linked to narcolepsy include variants of cathepsin H (CTSH, which encodes pro-cathepsin H, which processes peptides and is then presented by MHC class II on dendritic cells) and tumor necrosis factor ligand superfamily member 4 (TNFSF4, which regulates immune cell fate). IL10RB–IFNAR1 (the IL-10 and interferon receptor gene region), ZNF365 (that encodes the transcription factor ZNF365), and P2RY11 (encodes P2Y purinoceptor 11) and the chemokine receptor CCR1–CCR3 region are all linked to narcolepsy. Surprisingly, very rare mutations have been discovered in a small number of narcolepsy patients, implying that the disorder has its own pathogenesis. A single mutation in the hypocretin gene, which encodes hypocretin, has been explored, as has a mutation in the MOG gene, which encodes myelin oligodendrocyte glycoprotein, in a single pedigree with familial narcolepsy [7]. As a result, in addition to traditional HLA haplotypes, new polymorphic associations are being discovered in order to uncover the pathogenesis of narcolepsy and treat it with specific diagnostics.

### 3.3. Environmental Factors

Environmental factors have been identified as factors in the etiopathogenesis of narcolepsy, in addition to genetic factors. Monozygotic twin studies reveal a 25–31% narcolepsy concordance rate, demonstrating the disease’s multifactorial pathology [28]. The nature of possible environmental triggers for narcolepsy is largely unknown. TBI, stroke and a disrupted sleep–wake cycle are the nonspecific environmental factors most likely to be linked to the onset of the disease in a broader sense, as all of these could transiently or permanently modulate hypocretin levels [29]. Recent studies have shown a link between narcolepsy and streptococcal infection [30,31,32,33,34], seasonal influenza, pandemic A/H1N1 2009 influenza vaccination [35,36], and exposure to insecticides, format change weedicides and heavy metals [16,37]. One of the strongest pieces of evidence comes from the associations between Pandemrix^®^ vaccination and the onset of narcolepsy [36]. After a traumatic brain injury, narcolepsy may develop [38]. In addition, other environmental triggers influence the development of narcolepsy as novel unknown environmental factors may also cause narcolepsy in the future, so it is critical to investigate those factors from time to time [6].

## 4. Inflammatory Response

Anatomical studies have demonstrated that hypocretin-immunoreactive neurons are localized within the hypothalamus [39]. While working with their respective groups, Peyron et al. [40] and Thannickal et al. [41] discovered a significant global loss of hypocretin neurons in the brains of narcolepsy patients with cataplexy. Human narcoleptics have shown a reduced number of hypocretin neurons in the hypothalamus [40]. Important studies on animals reported that, unlike in other animals, hypocretin deficiency in cats is caused by the loss of hypocretin neurons in the dorsolateral hypothalamus, not by hypocretin gene mutations [42]. Overall, these findings suggest that in narcolepsy patients with cataplexy, inflammatory responses may play a role in hypocretin neuron elimination [16]. Several studies have linked narcolepsy with cataplexy to differential expression of cytokines linked to chronic inflammation. Chronic inflammation has a negative impact on T cells, including Tregs, and is seen in cataplexy patients shortly before the onset of narcolepsy [43]. Changes in the expression of immunomodulatory cytokines have been linked to narcolepsy, but it is unclear whether these changes are caused by a predisposing factor for narcolepsy or by a disease or its comorbidities such as diabetes, obesity or stroke. TNF injections into the brain stimulate NREM sleep while inhibiting spontaneous sleep [44], which could explain EDS in narcolepsy patients. Sleep disturbances are known to increase IL-6, and sleep deprivation disrupts the regulation of IL-6 and TNF- [45,46,47]. Extended daytime sleepiness elevates IL-6 and TNF-, which suggests that a disrupted sleep–wake cycle modulates cytokine profile in narcolepsy patients [48,49,50]. Furthermore, polymorphisms in the TNF-promoter gene have been proposed as a possible cause of increased TNF-secretion in narcolepsy patients [51]. Increased levels of cytokines/chemokines in narcolepsy patients are also thought to be a result of microbial infection or vaccine immunization [52]. When narcoleptics were compared to controls, Mohammadi et al. [53] found higher plasma levels of IL-6 and TNF-, an insignificant difference in serum IL-6 and TNF- levels, and lower CSF IL-6 levels. In addition, narcolepsy patients’ plasma and CSF levels of IL-8 (chemotactic factor) and IL-10 (anti-inflammatory cytokine) are unchanged when compared to controls. The inconsistency of inflammatory cytokine results in narcolepsy patients explains the limited neuroinflammatory response in the lateral hypothalamus, which may make cytokine concentration measurements in CSF or serum difficult [54]. In rats, IL-8 administered intracerebroventricularly promoted NREM sleep [55]. Future research should focus on the role of inflammation responses in narcolepsy and the most cost-effective anti-inflammatory drugs for narcolepsy treatment [53,55,56,57].

### 4.1. Autoimmune Hypothesis

**Figure 1:**
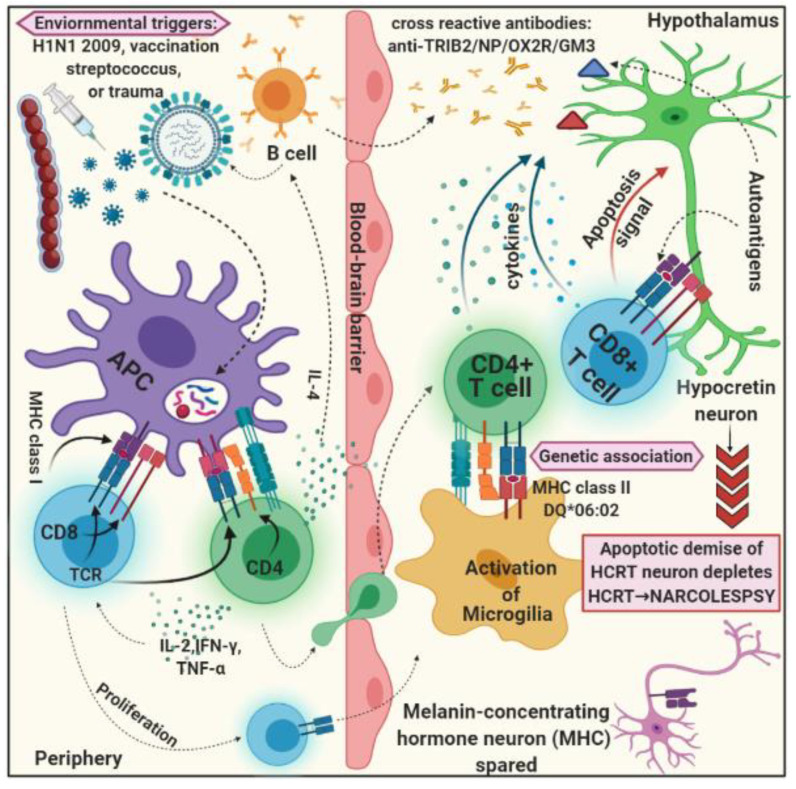
Schematic figure represents the possible mechanism for the autoimmune hypothesis for narcolepsy with cataplexy. Several environmental triggers are capable enough for molecular mimicry and elicit an immune response against microbiological/environmental factors. The microbiological-specific immune cells can cross-react with autoantigens and can activate autoimmune responses against hypocretin-secreting neurons in the hypothalamus, where the melanin-concentrating hormone is relatively spared.

The exact pathological mechanism that causes the death of hypocretin neurons in most narcolepsy patients with cataplexy is unknown. The removal of hypocretin neurons is very selective, sparing the melanin-concentrating hormone-releasing neurons found in the lateral hypothalamus, supporting the popular autoimmune hypothesis for narcolepsy disease pathogenesis [58,59]. The strong link between the HLADQB1*06:02 allele and polymorphisms in other immune-related genes and the immunological-autoimmune response in narcolepsy patients with cataplexy support the autoimmune hypothesis (Figure 1). Some studies have found a seasonal pattern in narcolepsy with cataplexy disorder [27], which could indicate infection triggers. Despite this sliver of evidence for an autoimmune mechanism underpinning narcolepsy with cataplexy disease, the autoimmune hypothesis has no direct support. There are still many pieces of evidence needed to prove that an autoimmune response is a central pathological mechanism in narcolepsy with cataplexy disorder. As a result, no definite signs of inflammatory processes in the CNS of narcolepsy patients with cataplexy and rising in the presence of other autoimmune disorders in narcolepsy patients have been consistently observed [40,60,61]. Surprisingly, no improvement in clinical symptoms or change in disease course has been observed following the administration of immunomodulatory therapy close to disease onset [62,63,64], with the exception of a single exceptional case report [7,65].

### 4.2. Autoantibody and T Cell Laboratory Findings

In addition to HLA genes, non-HLA genes have been shown to have a genetic link to narcolepsy predisposition. Polymorphisms in specific T cell receptor loci have been linked to narcolepsy [27,66]. As a result, T cells’ apparent role in the pathophysiology of narcolepsy with cataplexy is unequivocally emphasized by their involvement. The specific HLA–TCR interactions found in narcolepsy patients with cataplexy support the autoimmune hypothesis that T cells mediate selective irreversible elimination of hypocretin neurons [67]. In order to exploit autoreactivity as a mechanism underlying hypocretin deficiency in narcolepsy, autoantibodies must be searched in order to validate the direct evidence for the autoimmune hypothesis. Autoantibodies, on the other hand, have been overlooked in a few notable studies [68,69,70]. In contrast, several studies have discovered that the TRIB-2 protein (tribbles homolog 2), which is produced by many cells including hypocretin neurons, is a putative target for autoantibodies in narcolepsy patients with cataplexy one year after onset. In mice with passively transferred TRIB2 autoantibodies, narcolepsy with cataplexy phenotype can be seen [43,67].

Patients with autoimmune uveitis have TRIB2 autoantibodies as well [71,72,73]. Previous research reports have supported the hypothesis that there are low levels of serum TRIB2 autoantibodies in HLA-DQB1*06:02-positive young narcolepsy patients with cataplexy, which they linked to influenza pandemic vaccination and A/H1N1 antibodies in 2009 [74,75,76,77,78,79,80]. This discovery confirms that T cells can infiltrate the hypothalamus, but it disproves the autoimmune hypothesis due to their variable autoreactivity. Anti-Ma antibodies, on the other hand, have never been found in cataplexy patients with narcolepsy [81]. Fontana et al. [82] proposed that autoantigen-specific CD4+ T cells or superantigen-stimulated CD8+ T cells activate microglia/macrophages, which then destroy hypocretin neurons through immune-mediated destruction. Lecendreux and colleagues [43] investigated overall immune system activation in blood samples of pediatric narcolepsy patients with cataplexy. Regarding the phenotypic changes in T cells, elevated levels of activated memory effector CD4+ T cells were found to be associated with a higher absolute count of activated CD4+ T reg cells with a high frequency. In narcolepsy patients, the increase in Treg could be an attempt to reduce inflammation and restore homeostasis. Lecendreux and colleagues were the first to report the activated phenotype and changes in Treg frequency, and this finding strongly suggests that Tregs are also induced in narcolepsy with cataplexy as part of an autoimmune response [43]. According to this theory, in narcolepsy with cataplexy disorder, global but weak inflammation activates all T cell subsets, including Tregs, but Tregs are unable to maintain peripheral tolerance due to a qualitative defect, either in these Tregs or in pathogenic immune cells. Indeed, in T1D, Tregs have been shown to have a qualitative defect. Defective suppression is caused by a qualitative flaw or a dysfunctional Treg, which can lead to autoimmunity. Resistance to Treg-mediated regulation or Treg cell defect can be increased by changes in antigen presentation or the expansion of pro-inflammatory cytokines in a remodeled microenvironment (viz., Th 17 effector cells). As a result, it is an important aspect that should be investigated further in order to gain a better understanding of Treg plasticity and function in the context of autoimmune disorders [30]. Furthermore, Kornum et al. [83] found that polymorphisms in the P2RY11 gene affect the viability of natural killer, CD8+, and CD4+ T cells in narcolepsy patients with cataplexy. The disease-associated allele is linked to lower P2RY11 gene expression in CD8+ T lymphocytes and natural killer cells but not in other types of peripheral blood mononuclear cells. P2RY11 is a key regulator of immune cell survival, and it may play a role in narcolepsy with cataplexy by enhancing T cell survival [83]. TCR sequencing and deep sequencing of narcoleptic T cell subsets should be performed effectively to characterize a possible functional defect of Tregs, suppression and proliferation antigen-specific assays utilizing H1N1 peptides, as molecular mimicry has been hypothesized between hypocretinergic neurons and H1N1 virus [43,84]. Because the reactivity of T cells to hypocretins is unknown, T-cell transfer in narcolepsy animal models must be investigated [85,86,87,88,89,90,91,92,93,94,95,96].

## 5. Narcolepsy and Neuromediator Systems

Neuromediators involved in sleep–wake cycle regulation include hypocretins, dopamine, histamine acetylcholine, norepinephrine, serotonin, glutamate and GABA. Changes in these neuromediators’ levels can affect wakefulness, sleep and circadian rhythm, which is why they are clinically important. As a result, their roles in narcolepsy should be thoroughly investigated in order to determine the precise causative effect and to develop new therapeutic avenues that are relevant to the condition.

### 5.1. Hypocretinergic System

**Figure 2:**
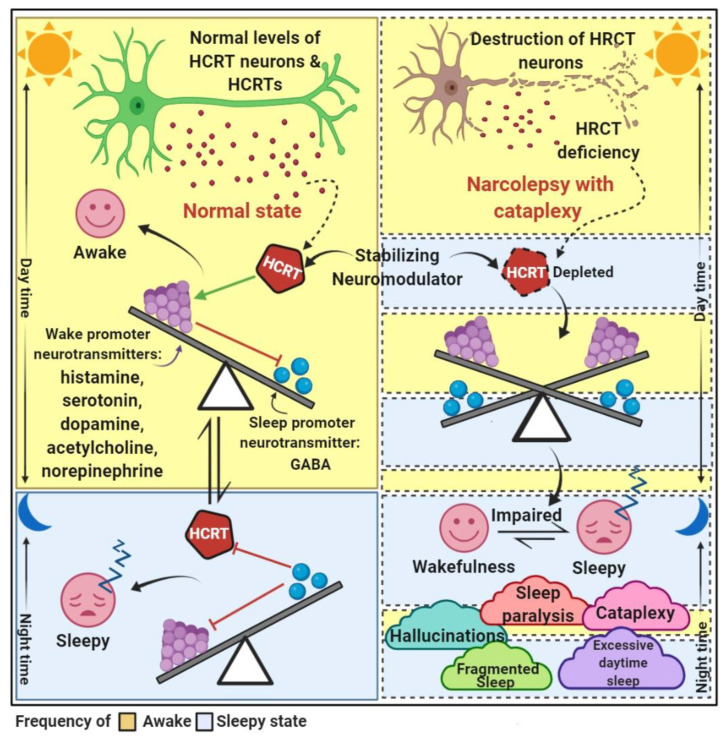
A representation of the normal narcolepsy state. Compared to normal individuals, narcolepsy patients with cataplexy have low levels of hypocretin (HCRT) due to a loss of hypocretin neurons. The flip-flop model of the sleep–wake state in normal conditions is balanced by stabilizing modulator hypocretin, while hypocretin deficiency unbalances the sleep–wake transition in narcoleptics. In normal individuals, hypocretin is thought to stabilize wakefulness during the day by both activating the ribocortex and stimulating the ascending arousal system to increase wake-promoting neuron activity. Sleep-promoting neurons inhibit both wake-promoting neurons and hypocretins. The wake-promoting and sleep-promoting neurons include excitatory neurotransmitters and inhibitory neurotransmitters. The main five symptoms of narcolepsy are manifested by narcolepsy patients.

The hypocretin system is made up of about 70,000 hypocretin-producing neurons in the human dorsolateral hypothalamus that project a virtual neural axis. Hypocretin neurons are a type of neuron that aids in the maintenance of the sleep–wake cycle. Hypocretins are neuroexcitatory peptides that are encoded by a hypothalamic-specific transcript and act as agonists of two G-protein-coupled receptors independently [87]. Hypocretin neurons produce two types of excitatory neuropeptides: hypocretin 1 (orexin-A) and hypocretin 2 (orexin-B). The tuberomammillary nucleus (histaminergic neurons), locus coeruleus (norepinephrine neurons), raphe nucleus (serotonergic neurons), laterodorsal tegmental nuclei (acetylcholine) and ventral tegmental areas (dopaminergic neurons) form an extensive network of projections throughout the brainstem [16,97,98,99]. Cataplexy is caused by hypocretin deficiency, which disables the motor excitatory systems and inhibits the motor inhibitory system in the brainstem. Depletion of hypocretin neurotransmission can increase sleepiness by disabling the cholinergic and aminergic arousal systems or disinhibiting the forebrain’s hypnogenic systems [97]. Hypocretin is a neuromodulator that keeps excitatory and inhibitory neurotransmitters such as histamine, serotonin, dopamine, acetylcholine, and norepinephrine in check. Hypocretin is a neurotransmitter that controls sleep, wakefulness, autonomic and energy homeostasis, food intake, consummatory and reward-related behaviors, pleasure-seeking behavior and emotional processing, among other things [16,87,99] (Figure 2).

### 5.2. Dopaminergic System

Muscle atonia during cataplexy and REM-sleep atonia are both shown to be a multistep process involving brainstem circuits that are shared by both. Inadequate activation of D1- and D2-like receptors may result in sleep attacks and cataplexy, respectively, emphasizing the importance of the dopaminergic pathway in narcolepsy [100]. The reintroduction of hypocretin into a specific brain region produces unexpected results. Hypocretin input in the locus coeruleus improves fragmented sleepiness in hypocretin receptors 1 and 2 double-knockout mice, whereas dorsal raphe specific to the serotoninergic pathway can prevent cataplexy, and activation of the GABAB pathway in hypocretin-neuron-deficient mice significantly alleviates cataplexy [101].

### 5.3. Histaminergic System

Histaminergic neurons, like hypocretin neurons, play an important role in promoting and stabilizing wakefulness by activating cortical neurons and wake-promoting neurons in the hypothalamic tuberomammillary nucleus. Hypocretin and histamines are neurotransmitters that work together to keep us awake and alert throughout the day. Histamine neurons either directly or indirectly inhibit non-REM sleep-promoting neurons by reinforcing the activation of wake-promoting neurons [89,102,103]. The histaminergic system has gained interest in sleep–wake regulation research in recent years, owing to the 60–80 percent augmentation of histaminergic neurons in the brains of narcolepsy and cataplexy patients [104,105]. Despite an increase in the number of histamine neurons in narcolepsy patients, histamine and tele-methylhistamine levels in the CSF are not useful biomarkers for narcolepsy [7].

## 6. Clinical Features

For people with narcolepsy, the persistent sleep–wake state instability can result in a wide range of unpredictable symptom manifestations throughout the day. Excessive daytime sleepiness (EDS), cataplexy, sleep paralysis, vivid hallucinations and disrupted nocturnal sleep are the five main symptoms of narcolepsy [106]. These symptoms can be debilitating and make daily life difficult. Hypothalamic hypocretin neurons are critical for ensuring the predictable timing and stable boundaries required for normal sleep–wake cycles. Inadequate activation of histamine and other wake-promoting neurons results in insufficient inhibition and intermittent activation of REM and non-REM sleep-promoting neurons during the day, allowing elements of REM sleep to intrude into wakefulness or REM sleep at the wrong time. This atypical instability in sleep–wake rhythm promotes frequent and unpredictable transitions between sleep–wake states, as well as rapid transitions to REM sleep, resulting in daytime naps or non-REM sleep at the wrong time, as well as rapid transitions to REM sleep or REM at the wrong time. Cataplexy and sleep paralysis can result from REM sleep-associated muscle atonia intruding into wakefulness at sleep–wake transitions. Cataplexy refers to brief episodes of muscle weakness or loss of muscle tone that are usually triggered by strong emotions or specific circumstances while awake, whereas sleep paralysis is often a frightening inability to speak, move limbs or open one’s eyes for several moments to several minutes immediately after awakening or falling asleep [107].

In narcolepsy, excessive daytime sleepiness (EDS) is caused by impaired alertness and neurocognitive functioning, which insufficiently activates wake-promoting neurons and weakly inhibits non-REM sleep-promoting neurons, resulting in an unpredictably sleepy state throughout the day, exacerbating EDS and sleep–wake state instability. Sleepiness or drowsiness is the most difficult symptom to manage. People with narcolepsy may fall asleep unexpectedly, such as while sitting in class or working at a computer. However, their sleep duration in a 24 h period is not significantly longer than that of normal people. Most people with this disorder believe that short 15 min naps improve their alertness for few hours. This conviction implies that the unusual excessive daytime sleepiness is caused by defects in the cerebral circuits that normally induce complete alertness in the natural state, rather than by insufficient or inadequate sleep. Healthy people sleep by going through a sleep cycle that lasts an hour or more before reaching the rapid eye movement (REM) sleep stage, which is characterized by dreams. On the contrary, narcoleptics fall asleep quickly, within up to five minutes, and enter the REM phase quickly, resulting in a very realistic dream, even in short naps. Narcolepsy is a neurosynaptic disorder that impairs the ability to regulate sleep and wake cycles. As a result, people with narcolepsy have shaky boundaries between wakefulness and sleep, causing them to fall asleep quickly and even encouraging frequent lapses into sleep [108,109].

## 7. Pathophysiology and Links with Other Diseases

The REM sleep and hypocretinergic system were discovered around the turn of the millennium, and sleep research has been burgeoning since then. The discovery of hypocretins demonstrated the importance of these neurons in physiological sensory functions, as well as their compelling role in narcolepsy pathology. The pathophysiology of narcolepsy is heavily influenced by genetic and environmental factors, and the exact pathogenesis is unknown. The most likely cause of narcolepsy associated with cataplexy is the destruction of hypocretin-producing neurons and the resulting low level of hypocretin (Narcolepsy type 1). NT1, rather than narcolepsy without cataplexy (Type 2), is the most widely studied [59]. Narcolepsy is widely defined as the death of selective hypothalamic hypocretin peptide-producing neurons. The HLA class II molecule mediates macrophage and microglia activation, which results in the release of neurotoxic pro-inflammatory molecules such as Fas ligand, tumor necrosis factor, interleukin-1, glutamate, quinolinic acid, reactive oxygen species and nitric oxide, which may destroy hypocretin neurons. Quinolinic acid has been shown to have destructive sensitivity. In line with this, Katsuki et al. [110] demonstrated that NMDA has a cytotoxic effect on hypocretin and melanin-concentrating hormone (MCH) neurons [92]. The investigation of endogenous glutamate receptor agonists (i.e., kainic acid) and glutamate transporter blockers (i.e., hydroxyapatite) reveals that quinolinic acid acts as an endogenous excitotoxin that can cause specific loss of hypocretin neurons, whereas activating NMDA receptors spares MCH immunoreactive neurons. The excitotoxic consequence is the irreversible elimination of hypocretin neurons and the transient disappearance of immunoreactivity for MCH, which occurs primarily as a result of neuropeptide depletion from respective neurons released in response to excitatory stimuli due to compromised peptide synthesis under excitotoxic insults. However, many other putative intracellular mechanisms control excitotoxic effects on central neurons, and now, every possible pathophysiological pathogenesis remains to be a determinant of hypocretin neurons’ and MCH neurons’ vulnerability to excitotoxicity [110]. In narcoleptic mice, induced gene inactivation of the prepro-hypocretin (HCRT) or hypocretin gene receptors, or genetic deletion of hypocretin neurons, results in insufficient hypocretin neurotransmission and reasonable catalytic attacks [111].

### 7.1. Narcolepsy and Diabetes

The link between narcolepsy and diabetogenic hyperinsulinemia was first discussed five decades ago [112]. Narcolepsy is strongly associated with an increased BMI and the prevalence of type 2 diabetes [55]. Given that hypocretin regulates food intake and energy homeostasis, it appears that narcolepsy and metabolic disorders are linked. Diabetes mellitus, a well-known metabolic disorder, is strongly linked to narcolepsy. As prominent putative mechanisms for this association, changes in food intake, imbalanced energy consumption, glucose tolerance, and insulin sensitivity, as well as inflammation and genetic factors, have been proposed. The primary cause of narcolepsy, hypocretin deficiency, is associated with increased food intake and decreased basal metabolic rate (BMR), both of which lead to obesity. The anti-apoptotic effect of hypocretin on pancreatic beta-cells raises peripheral insulin sensitivity while decreasing lipolysis in adipose tissue, raising the risk of obesity and type 2 diabetes (T2D) in narcoleptic patients. Autoimmunity and inflammation are the primary pathomechanisms shared by narcolepsy and type 1 diabetes (T1D). Indeed, the main predisposing HLA gene for narcolepsy, DQB1*0602, also acts as a protective factor for T1D [113], whereas CTSH mutations are an odd factor for both T1D and narcolepsy. As a result, these two genes provide the most compelling genetic evidence for the link between narcolepsy and T1D. Narcolepsy is also linked to gestational diabetes mellitus (GDM), as it can lead to glucose intolerance, type 2 diabetes and obesity. GDM patients typically have low serum hypocretin levels, which are associated with low fasting insulin and high fasting glucose levels. Furthermore, the increased prevalence of obesity in narcolepsy patients was quickly identified as a major risk factor, emphasizing the link between narcolepsy and diabetes mellitus [114]. The effect of exercise improving narcolepsy is contradictory, but overweight or obese patients with fibromyalgia and narcolepsy should be encouraged to lose weight [115,116]. Patients with narcolepsy and cataplexy appear to be at increased risk for diabetes because they frequently exhibit obesity, hypertension and T2D, and they share genetic and environmental risk factors with T1D as well [43,117,118]. Hypocretin neurons are found primarily in the posterior and lateral hypothalamic nuclei, as well as in the anterior hypophysis, adrenal medulla and enteric nervous system and throughout the pancreatic neuro-enteric system [119]. Hypocretin neuropeptides function by interacting with two G-coupled hypocretin receptors, type 1 having a high affinity for hypocretin A and type 2 having the same affinity for both hypocretin A and B. The anterior pituitary gland, the adrenal medulla, the spinal ganglions, and a variety of other tissues such as adipose, gut, pancreatic, adrenal gland and testis tissues all contain hypocretin receptors [120]. Endogenous signaling pathways for hypocretin are as diverse as its expression sites. Gq-phospholipase C is an omnipresent hypocretin signaling mediator in a variety of cell types, followed by downstream cascades of DAG and IP3-Ca2+ release, and modulates excitation/inhibition balance as well as gene expression in a variety of cells. The activation of Gi-adenylyl cyclase and the phosphatidylinositol 3-kinase (PI3K)-protein kinase C (PKC), both of which result in a high Ca^2+^ influx, are two of the most common pathways mediating hypocretin’s central effects, particularly in the hypothalamic nuclei [120,121,122,123,124,125,126,127,128,129,130].

### 7.2. Narcolepsy and Ischemic Stroke

**Figure 3:**
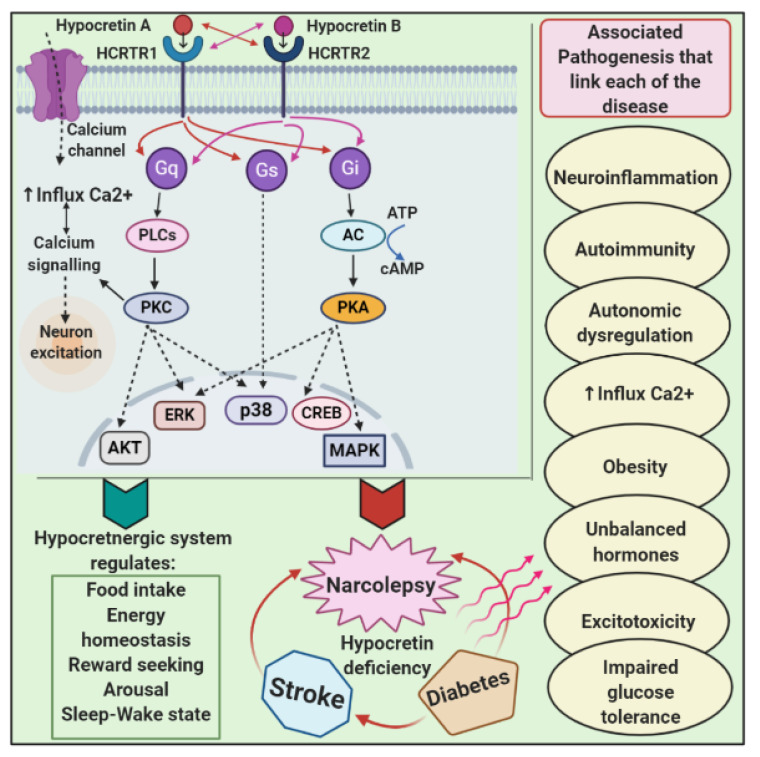
Diagrammatic representation of the pathological linking between narcolepsy, diabetes and stroke. Hypocretin A and hypocretin B are two types of hypocretins that mediate narcolepsy’s functional role via G protein coupled receptor-hypocretin receptors type 1 and type 2. The downstream cascade activates various transcription factors and implicates in the regulation of several processes. The main common pathogenesis for narcolepsy, diabetes and stroke is hypocretin deficiency. There are numerous underlying pathophysiological mechanisms that are associated that link these diseases.

Ischemic stroke is a type of cerebrovascular disease with a high mortality and disability rate around the world [131]. Brain tissue apoptosis and necrosis are caused by insufficient blood supply and a lack of oxygen [132,133,134]. Diabetic ischemic stroke has a multifaceted and complex pathophysiologic mechanism that includes excitotoxicity, endothelial dysfunction, disrupted energy homeostasis, oxidative stress, inflammation, disruption of the blood–brain barrier, atherosclerosis and other factors [135,136,137,138]. Several studies have demonstrated that hypocretins are an effective neuroprotective agent in cerebral ischemic insult and ischemia-reperfusion injury. Hypocretin possesses antioxidant, anti-inflammatory and anti-apoptotic properties, allowing the hypocretinergic system to perform cytoprotective functions, activate proliferation and normalize metabolism [139]. Patients with cerebral infarction or ischemic injuries have low levels of CSF hypocretins [140,141,142,143]. Furthermore, CSF hypocretins are reduced in patients with subarachnoid hemorrhage (SAH) for 10 days after the triggering event. CSF hypocretin levels are higher in patients who do not develop DID from complications of delayed ischemic neuronal deficit (DID) caused by symptomatic vasospasm in SAH patients. It is hypothesized that decreased hypocretin production in SAH and DID patients is associated with altered hypocretin signaling in response to ischemia [144]. Several rodent studies have revealed that hypocretin A strongly induces type 1 hypocretin receptor not only in neurons but also in astrocytes and oligodendrocytes, implying that type 1 hypocretin receptor is primarily associated with a cerebral ischemic insult [145]. Furthermore, increased expression of type 1 hypocretin receptor expression correlates with decreased hypocretin concentration in ischemic animal models’ cerebrospinal fluid. Notably, Irving et al. [45] found an increase in mRNA and protein levels of type 1 hypocretin receptor but not type 2 hypocretin receptor, as well as dynamic changes in CSF hypocretin A levels in rat ischemic cortex after permanent middle cerebral artery occlusion (MCAO). These findings imply that significant changes in hypocretin A and type 1 hypocretin receptors may play functional roles in neuronal damage caused by ischemic injury [144]. Furthermore, intracerebroventricular injection of hypocretin A reduces cerebral infarct volume and improves neural deficits caused by MCAO in mice [146]. The mechanism underlying hypocretin’s neuroprotective potential is most likely associated with a decrease in apoptotic cells and activation of HIF-1. The administration of an HIF-1 inhibitor, on the other hand, suppresses the stroke-induced increase in HIF-1 and reverses the cytoprotective effect of hypocretin [147]. Surprisingly, hypocretin promotes neuron survival in the rat cerebral cortex in a concentration-dependent manner. Furthermore, hypocretins have pro-survival properties due to decreased caspase-3 activity [148]. Hypocretin, according to Harada et al. [146], promotes the expression of brain-derived neurotrophic factor (BDNF) to enable neuroprotective functions and prevents neural damage in ischemic subjects. Hypocretin treatment altered the cytokine profile in hypocretin/ataxin-3 transgenic ischemic mice, specifically altering the expression of IL-6 and TNF at the mRNA level, implying that a chronic inflammatory response is also involved in this process [149]. According to these findings, the hypocretinergic system protects nerve cells from cerebral ischemia and ischemic-reperfusion injury by regulating anti-apoptotic and inflammatory responses. As a result, hypocretin deficiency may be responsible for clinical manifestations in stroke patients and may result in narcolepsy disease [150]. Future advances in our understanding of the common pathological features of stroke, diabetes, and narcolepsy, as well as the mechanisms of hypocretin-associated protection, may point to the possibility of using hypocretins to treat these diseases [151] (Figure 3).

### 7.3. Narcolepsy and Alzheimer’s Disease (AD)

Alzheimer’s disease (AD) is a neurodegenerative disease that causes progressive cognitive decline and behavioral impairment. The neuropathological hallmarks of AD include extracellular-amyloidal deposition and the formation of neurofibrillary tangles caused by tau protein hyperphosphorylation, undermining neuronal and synaptic functions. Some studies have concluded that Alzheimer’s disease is associated with hypocretinergic neuronal loss as well as impaired hypocretin neurotransmission, implying that the hypocretin neuronal system plays an important role in the pathogenesis of Alzheimer’s disease [151]. Fronczek et al. [152] quantified hypocretin neurons and measured hypocretin levels in the cerebrospinal fluid (CSF) of post-mortem hypothalamus of AD patients and found a 40% decrease in hypocretin neurons and a 14% decrease in CSF hypocretin-1 levels. Increased CSF-hypocretin levels, on the other hand, have been reported in some Alzheimer’s disease patients [153,154,155,156,157,158]. As low hypocretin decreases amyloid release, NT1 patients who have lost most of their hypocretin-containing neurons, seem to have a lower risk of developing AD [40,41]. Scammell et al. [159] suggested that chronic loss of hypocretin signaling provides no protection against Alzheimer’s disease, implying that normal levels of hypocretin are not required in the pathogenesis of the disease [160].

### 7.4. Narcolepsy and Parkinson’s Disease

Parkinson’s disease (PD) is characterized by motor-related symptoms such as tremors, rigidity, slowness of movement and difficulty with walking and balance. Comorbid conditions in PD individuals include narcolepsy-like sleep patterns. The intersecting sleep symptoms of both conditions include excessive daytime sleepiness, hallucinations, insomnia and falling into REM sleep more quickly than an average person [161]. These sleep symptoms are also described in patients suffering from the sleep–wake disorder, i.e., narcolepsy. The International Classification of Sleep Disorders (ICSD-2) narcolepsy criteria use a number of markers for diagnosis, of which the lack or deficiency of cerebrospinal fluid (CSF) hypocretin-1 levels is a key marker. Hypocretin neurons prominently located in the lateral hypothalamus and perifornical nucleus have been proposed to interact with mechanisms involving sleep and arousal [162,163]. Low hypocretin-1 levels in the CSF have been shown to correlate with hypothalamic hypocretin cell loss in narcolepsy and other forms of hypersomnia; therefore, it has been proposed that degenerative damage to hypocretin neurons (such as in PD) may be detected by low CSF hypocretin-1 concentrations and may also explain the sleep symptoms experienced by some PD patients [163]. The neuropathology of the hypothalamus in Parkinson’s disease indicates a massive hypocretin loss, probably underlying the narcolepsy phenotype [164]. The benefit of the new, 24 h long acting ropinirole and transdermal rotigotine on sleep and sleepiness is modest. Eventually, the dopamine release in the mesocorticolimbic pathway is increased during rapid eye movement sleep, supporting its role in dopaminergic-induced vivid dreams [164,165,166]. Various sleep-related problems, for example, insomnia and symptoms of rapid eye movement behavior disorder, are common in patients with Parkinson’s disease (PD) [165]. Symptoms of rapid eye movement behavior disorder were associated with symptoms of narcolepsy, including symptoms of cataplexy [167,168]. Chunduri and colleagues [161] identified genetic signatures that link PD with its comorbid disorders and narcolepsy, including the convergence and intersection of dopaminergic and immune system related signaling pathways. These findings may aid in the design of early intervention strategies and treatment regimes for non-motor symptoms in PD patients as well as individuals with narcolepsy.

## 8. Diagnosis and Treatment

The most likely causes of narcolepsy are an autoimmune response against hypocretin-secreting neurons and low CSF-hypocretin levels (110 pg/mL) [16]. Accurate diagnosis is required for effective treatment [169]. Cataplexy is a prominent feature of narcolepsy, but it is not a typical symptom limited to narcolepsy patients because it occurs in fainting, malingering and migraine patients as well. Multiple polysomnography or a mean latent sleep test (MLST) is commonly used to diagnose narcolepsy. While a person is sleeping, both tests use physiological measures such as EEG and an electrocardiogram. MSLT is a standard test that has a sensitivity of 78% and a specificity of 93% for narcolepsy diagnosis. According to the ICSD-3 criteria, an MSLT test result of less than 5 min indicates narcolepsy. Comorbidities can influence the MLST result: a decrease in sleep time can be caused by depression or stress, and tension can increase sleep time; therefore, it is critical to interpret MLST results carefully [169]. The quantification of low hypocretin levels in CSF is another narcolepsy diagnostic test with high sensitivity and specificity, but there is no standard method for it. Indeed, a decreased level of CSF hypocretin has been found in some diseases, including Prader–Willi syndrome [170,171] and multiple sclerosis [172]. Furthermore, some narcoleptic patients have a normal range of hypocretin [173,174]. Filardi and colleagues [175] proposed the use of actigraphy for the screening of narcolepsy, as actigraphy provided a reliable objective measurement of sleep quality and daytime napping behavior able to distinguish central disorders of hypersomnolence and in particular narcolepsy type 1.

Despite the lack of a standard measure, the low range for CSF hypocretin for narcolepsy diagnosis must be fixed in order to be correctly interpreted. Furthermore, because the age of narcolepsy onset has a bimodal distribution in terms of childhood and adolescence, age is another important criterion to be considered for the diagnosis and confirmation of the narcolepsy disease [34,65,176]. As a result, more dependable and precise methodologies for correct diagnosis and confirmation of narcolepsy are required [24]. Because no cure for narcolepsy has been discovered to date, almost all established treatments are primarily symptomatic. Nonpharmacological treatment may alleviate some of the symptoms of narcolepsy, taking scheduled naps reduces excessive daytime sleepiness both subjectively and objectively, and a low-carbohydrate, high-protein diet can improve wakefulness. However, no randomized controlled trial has been conducted to support the efficacy of nonpharmacological therapy. Furthermore, treating narcolepsy with nonpharmacological therapy alone is ineffective. According to a report published by the American Academy of Sleep Medicine, “scheduled naps can be beneficial in combating sleepiness but rarely suffice as primary therapy for narcolepsy” [167]. The primary treatment option for excessive daytime sleepiness is pharmacological therapy [16]. Current narcolepsy treatments provide some symptomatic relief at the expense of significant side effects. The exact nature of altered functions and effects in type 2 hypocretin receptor mutant dogs is still unknown. If the mutations result in a reduced functional response to hypocretin, the administration of the hypocretin receptor may alleviate narcolepsy symptoms. Similarly, hypocretin administration can reverse the disastrous effects of hypocretin deficiency in knockout mice. Similarly, if a similar pathogenesis occurs in humans, the same treatment for the aforementioned hypocretin agonists or hypocretin administration may be effective. Nonetheless, because hypocretin has been shown to regulate eating behavior, neuroendocrinology, arousal behavior, reward-seeking behavior and pain behavior, major brain systems are likely to be modulated when hypocretins are introduced. The potential effects on the immune system should also be considered [81,177].

### 8.1. Future Therapeutics

Hypocretin-1 intranasal administration and transplantation of neonatal hypothalamic stem cells into the brainstem are both promising therapeutic approaches. However, intracerebroventricular (ICV) administration of hypocretin-1 restores fragmented sleep patterns to normal levels, which improves wakefulness and reduces cataleptic episodes [178], but not in hypocretin-2-mutated dogs. Steroids, plasmapheresis and intravenous immunoglobulin are short-term immunomodulatory treatments used to treat autoimmune-induced narcolepsy with cataplexy. Clinical trials for histamine H3 receptor antagonists are currently underway for a variety of central nervous system disorders, including narcolepsy. Following acute administration, these agents can increase wakefulness in cats and rodents, but their effects after repeated dosing have not been reported. In addition, thyrotrophin-releasing hormone and the nicotine patch are both promising new treatments for narcolepsy. Future research is required to validate and establish future treatment options as effective therapeutic strategies for narcolepsy [179,180,181,182,183,184,185,186]. Table 1 contains a list of potential therapeutics that may help with narcolepsy symptoms.

### 8.2. Prospects for Future Research

To better understand the pathological mechanisms of hypocretin neuron loss in narcolepsy, it is necessary to expand our understanding of hypocretin nerve cell neurobiology. Because of significant breakthroughs in induced pluripotent stem cell (iPSCs) technology, in vitro neurological disease modeling for preclinical research has greatly improved. Adult differentiated somatic cells (i.e., human fibroblast cells) are reprogrammed using iPSC technology to generate pluripotent stem-cell-like cells [187]. In addition to autogenous cell replacement, iPSC technology allows for in vitro disease modeling and the discovery of either generalized or personalized drugs [188]. Researchers are unable to ensure that susceptibility genes of narcolepsy with cataplexy act cell-autonomously in hypocretin neurons or cell-non-autonomously in the immediate hypothalamic environment using current technology and knowledge. However, these mechanisms do not explain the actions of the immune-related predisposition genes DQB1*06:02 and TCRA. To identify these contributing factors, direct isolation of immune cells or iPSCs from narcolepsy patients, as well as a relevant cell-type differentiation protocol, will be required. Surprisingly, the interactions between hypocretin neurons and their general and immune environments are presumably genotype-dependent, and thus the iPSC approach can help to elucidate it. Nonetheless, the establishment of an in vitro narcolepsy model appears to be extremely difficult at the moment, but hypocretin neurons or hypothalamic cells from patients would be extremely important. A deep understanding of effector immune mechanisms associated with injury or lesion formation is critical for the development of demanding and significant immune therapies.

**Table 1:** Potential therapeutics that may improve narcolepsy symptoms.

Sr No.	Class	Drug Candidate	Mode of Action	Improve Narcolepsy Symptoms
1	Stimulants [189,190,191,192,193]	Modafinil	Blocks several monoaminergic transporters and inhibits dopamine reuptake transporter.	Potential to cause fatal hepatotoxicity; no longer recommended.
-	-	Armodafinil	(R)-enantiomer of modafinil more-potent and long-lasting	Treats excessive daytime sleepiness.
-	-	Methylphenidate	Non-competitive dopamine reuptake blocker and, to a lesser degree, a serotonin–noradrenaline reuptake blocker.	Second-line treatment for excessive daytime sleepiness.
-	-	Dextro-amphetamine sulfate	Competitive dopamine transporter blocker that also blocks the vesicular mono amine transporter.	Third-line treatment for excessive daytime sleepiness.
-	-	Pemoline	Selectively blocks dopamine reuptake.	
-	-	Solriamfetol (JZP-11)	Inhibits norepinephrine-dopamine reuptake.	Treats impaired wakefulness and excessive sleepiness.
2	Sodium salt of γ-hydroxybutyrate (GHB; a neurotransmitter that related to γ-aminobutyric acid (GABA) [194]	Sodium oxybate	GABAB receptor agonist that activates the GABA type B receptor and possibly its own specific GHB receptor.	First-line treatment for cataplexy. Improves “qualitative wakefulness”—fewer nightly awakenings, reduces NREM stage 1 sleep and increases slow-wave sleep, decreases arousals, and has a variable effect on latency and amount of REM sleep. Alleviates sleep paralysis, hypnagogic hallucinations.
3	Antidepressant [16,162,195,196,197,198,199]	Venlafaxine, duloxetine, reboxetine, and viloxazine (SNRI), atomoxetine (SNRI)	Blocks serotonin–noradrenaline reuptake pumps.	First-line off-label to treat cataplexy. Improves excessive daytime and night-time sleepiness. Second line to treat cataplexy.
		Clomipramine, imipramine (TCA)	Mono-aminergic reuptake inhibition, inhibit the reuptake of catecholamines.	It increases muscle tone and suppresses REM sleep. Second line to treat cataplexy.
		Selegiline (MOAI)	Monoamine oxidase type B inhibitor.	Preferred initial choice for treatment of excessive daytime sleepiness. Can be used to treat cataplexy.
		Fluoxetine, Femoxetine, Citalopram (SSRI)	Blocks serotonin reuptake pumps.	Second line or third line to treat cataplexy.
4	Psychoactive drugs [200,201]	Benzodiazepines and hypnotics, Triazolam, Ambien, clonazepam	Enhances the effect of the neurotransmitter gamma-aminobutyric acid (GABA) at the GABA_A_ receptor.	Second-line disrupted nocturnal sleep. Decreases arousal at night and sleep fragmentation. It improves cataplexy and sleep paralysis.
5	H_3_ blockers [202]	Pitolisant	histamine H3 receptor antagonists (inverse agonist).	Improves excessive daytime sleepiness. Not recommended in the newest guidelines.
6	Hypocretin replacement therapy or hypocretin receptor agonists [203]	-	HCRTR2 agonist-YNT-185, trace amine-associated receptor 1 (TAAR1) agonist.	Improves cataplexy, excessive daytime sleepiness, sleep paralysis and hypnagogic/hypnopompic hallucinations and suppress REM sleep.
7	Non-pharmacological therapy [203]	Sleep–wake schedules	-	Single scheduled daytime nap (of about 2 h) reduces the total amount of involuntary daytime sleep. Combined with stimulant treatment, two 15 min naps per day, and regular night-time sleep schedules, it has been shown to reduce subjective drowsiness and involuntary daytime sleep. Intensity of night-time NREM sleep is improved by daytime sleep restriction.
8	-	Psychosocial guidance	-	Helps in awareness and improves social problems associated with narcolepsy
9	-	Slow-wave sleep-enhancing treatments	-	Improves sleep

Given that an autoimmune response is an obvious pathogenesis of narcolepsy, iPSC technology is emerging as a therapeutic intervention; however, using transplantation of differentiated iPSCs can result in anamnestic autoimmune destruction of the therapeutic cells, as seen with islet cell transplantation for autoimmune type 1 diabetes. As a result, the development and application of patient-derived induced pluripotent stem cell systems can ensure the identification of novel potential targets for narcolepsy therapeutic intervention. Immunosuppressive therapies may become necessary in the case of autologous cell transplantation [55].

More research is needed to develop potential therapeutic strategies for narcolepsy and its incapacitating symptoms. Several large, randomized, placebo-controlled trials have demonstrated that modafinil and sodium oxybate are effective treatments for EDS associated with narcolepsy [19]. Traditional stimulants such as amphetamine, methamphetamine, dextroamphetamine and methylphenidate are cheap in generic form and widely used in clinical practice, but there is little high-level evidence from published studies. There is an urgent need for randomized trials that compare traditional stimulants to novel somnolytic agents in order to determine the relative efficacy and safety of these agents so that clinicians can appropriately choose between them and rationally prescribe them to individual patients. Furthermore, future research should focus on the development of new, more effective and well-tolerated therapies, as well as EDS primary prevention. Furthermore, despite extensive clinical experience, antidepressants are recommended to treat cataplexy, but they have been poorly validated in clinical trials. Randomized controlled trials of a wide range of antidepressants, particularly in comparison to the expensive but effective sodium oxybate, are desperately needed to assist clinicians in medication selection. Having said that, narcolepsy clinical trials must include children, the elderly, pregnant and nursing women, and other vulnerable populations [173].

## 9. Conclusions

Narcolepsy is a rare disease that has a negative impact on a person’s physical, emotional and social well-being. Symptoms of dysregulated REM sleep include cataplexy, sleep paralysis and hypnagogic hallucinations. The exact pathophysiology of narcolepsy is still unknown, despite decades of research. The selective destruction of hypocretin neurons is the most likely cause of narcolepsy with cataplexy. As the downstream cascade activates various transcription factors and is implicated in the regulation of several processes, it seems that the main common pathogenesis for narcolepsy, diabetes and stroke is hypocretin deficiency with a great number of pathophysiological mechanisms that are associated with those diseases. There have been significant advances in highlighting the pathogenesis of narcolepsy, with substantial evidence for an autoimmune response against hypocretin neurons; however, there are some gaps that need to be filled. To treat narcolepsy, more research should be focused on identifying molecular targets and novel autoantigens. In addition to therapeutic advances, standardized criteria for narcolepsy and diagnostic measures are widely accepted, but they may be reviewed and updated in the future with comprehension. Tailored treatment to the patient’s symptoms and clinical diagnosis and future treatment modalities with hypocretin agonists, GABA agonists, histamine receptor antagonists and immunomodulatory drugs should be aimed at addressing the underlying cause of narcolepsy.
